# Medical studies in times of a pandemic – concepts of digital teaching for Orthopaedics and Trauma at german universities

**DOI:** 10.1186/s12909-023-04213-4

**Published:** 2023-04-18

**Authors:** Anna-Maria Mielke, Mohamed Ghanem, David Alexander Back, Susanne Fröhlich, Stephanie Herbstreit, Ricarda Johanna Seemann

**Affiliations:** 1 Centrum für Muskuloskeletale Chirurgie, Charité - Universitätsmedizin Berlin, Corporate Member of Freie Universität Berlin, Humboldt-Universität zu Berlin, Berlin Institute of Health, Augustenburger Platz 1, 13357 Berlin, Germany; 2 Arbeitsgemeinschaft (AG) for Teaching at German Society for Orthopaedics and Trauma Surgery (DGOU), Berlin, Germany; 3 Arbeitsgemeinschaft (AG) for Digitalization at German Society for Orthopaedics and Trauma Surgery (DGOU), Berlin, Germany; 4 Department of Orthopaedic Surgery, Traumatology and Plastic Surgery, University Hospital Leipzig, Leipzig, Germany; 5 Dieter Scheffner Center for Medical Education and Educational Research, Charité - Universitätsmedizin Berlin, Corporate Member of Freie Universität Berlin, Humboldt-Universität zu Berlin, Berlin Institute of Health, Berlin, Germany; 6 Clinic for Traumatology and Orthopedics, Bundeswehr Hospital Berlin, Berlin, Germany; 7 Orthopaedic Clinic and Polyclinic, University Clinics Rostock, Rostock, Germany; 8 Department of Orthopaedics and Trauma Surgery, University Hospital Essen, Essen, Germany

**Keywords:** Digital Health, Undergraduate Medical Education, Digital competencies, Medical students, Survey, Ehealth, Medical education, Digital literacy, Pandemic, Covid-19, Digital Classroom

## Abstract

**Background:**

Due to the Covid-19 pandemic, on-site classroom teaching became limited at most German medical universities. This caused a sudden demand for digital teaching concepts. How the transfer from classroom to digital teaching or digitally assisted teaching was conducted was decided by each university and/or department individually. As a surgical discipline, Orthopaedics and Trauma have a particular focus on hands-on teaching as well as direct contact to patients. Therefore, specific challenges in designing digital teaching concepts were expected to arise. Aim of this study was to evaluate medical teaching at German universities one year into the pandemic as well as to identify potentials and pitfalls in order to develop possible optimization approaches.

**Methods:**

A questionnaire with 17 items was designed and sent to the professors in charge of organising the teaching in Orthopaedics and Trauma at each medical university. A differentiation between Orthopaedics and Trauma was not made to allow a general overview. We collected the answers and conducted a qualitative analysis.

**Results:**

We received 24 replies. Each university reported a substantial reduction of their classroom teaching and efforts to transfer their teaching to digital formats. Three sites were able to switch to digital teaching concepts completely, whereas others tried to enable classroom and bedside teaching at least for students of higher edcuational levels. The online platforms used varied depending on the university as well as the format it was supposed to support.

**Conclusion:**

One year into the pandemic significant differences concerning the proportions of classroom and digital teaching for Orthopaedics and Trauma can be observed. Simultaneously huge differences in concepts used to create digital teaching are present. Since a complete suspense of classroom teaching was never mandatory, several universities developed hygiene concepts to enable hands-on and bedside teaching. Despite these differences, some similarities were observed: the lack of time and personnel to generate adequate teaching material was reported as the leading challenge by all participants of this study.

**Supplementary Information:**

The online version contains supplementary material available at 10.1186/s12909-023-04213-4.

## Introduction

Advances in digitalization and digital transformation have a fair amount of influence on our daily lives. May it be for private or work-related use, they are also becoming relevant in terms of medical teaching [1–4]. For younger generations of medical students being born after 1982 and growing up in the age “4.0” they are essential parts of their daily lives [5]. Still, until recently online class concepts were rarely used to teach medical students [6].

The definition of digital or digitally assisted teaching includes all forms of teaching using electronic or digital media for presentation and distribution of study material or communication between people [7]. This means digital media may be used for teaching as well as exams at medical universities. However, the use of social communication tools, interactive media, and electronic exams at German universities is still quite inhomogeneous [8].

The Covid-19 pandemic represented and still represents a new challenge for students and teachers alike.

Medical studies for a medical degree strive to teach the required knowledge in the easiest, most effective and secure way as possible, using pre-defined study goals as guideline [9].

Starting April 1st 2020, amendments to the German regulations of undergraduate medical education were made to enable further teaching and exams under the new conditions of the Covid-19 pandemic. It allowed classroom teaching to be partially or completely replaced by digital teaching [10]. How the transfer from classroom to digital teaching or digitally assisted teaching was conducted was subject to each university and/or department individually. Still the transformation of teaching and study material was performed in a remarkably short period of time.

Orthopaedics and Trauma are medical specialties with increased focus on hands-on teaching as well as direct contact to patients to practise examination techniques. This specifically includes the preoperative planning of approach and placement of materials as well as the handling of various different materials (plates, screws, wires etc.) and tools used for osteosynthesis. Still, several publications over the past years were able to show successful approaches to integrate digital teaching at different universities [11–13].

With this study we tried to create an overview concerning the extent and forms of digital teaching for Orthopaedics and Trauma at German universities under current regulations for the pandemic. From this information and pre-existing literature, we hoped to identify challenges and to derive suggestions for established as well as new formats of teaching.

## Methods

### Study design

The authors developed a questionnaire to collect information on digital teaching currently used under Covid-19 regulations in Orthopaedics and Trauma at German universities. Furthermore, it included questions on potentials and pitfalls in (digital) teaching arising from the pandemic in retro- and prospective.

The questionnaire was then distributed via the German Association for Orthopaedics & Trauma (DGOU) and sent to the professors in charge of organising the teaching in this discipline at each medical university. Answers were collected from April 30th until October 15th and used as the foundation for our analysis and conclusion. All contributions were voluntary.

### Questionnaire

We developed a questionnaire with 17 items (open questions, multiple choice, likert scale) to examine following aspects:

- Degree course in the field of medical studies.
- Currently used teaching formats.
- Used teaching concept (classroom vs. online teaching, hybrid approach).
- Platforms used (e.g. Zoom, Microsoft teams).
- Amount of bed-site teaching and patient contact.
- Hygiene concept.
- Currently used exam formats and concepts.
- Voluntary classes in Orthopaedics & Trauma.

Additional interviews and open questions were used to gather opinions on potentials and pitfalls concerning the current teaching situation in this discipline. Suggestions for optimisation were gathered. Their answers were analysed and categorised by catchphrases as well as topics e.g., digital infrastructure, time management, staff, legal requirements etc. from four authors separately.

### Analysis

The voluntary answers to questionnaires were transferred electronically. Descriptive analysis of questionnaire answers was performed using Excel (Microsoft Inc., Redmond, WA, USA).

## Results

The questionnaire was sent out to 42 faculties, which are members of the DGOU. We received 24 replies (57%) to our questionnaire from 24 teaching coordinators for Orthopaedics, Traumatology, or both at 23 universities. Their number of students ranging from 180 to 933 per year (mean 336 ± 159). Several similarities were visible concerning teaching formats and their concepts, hygiene concepts, and identified challenges in medical teaching. The following table shows that all universities used digital teaching but the extent differed broadly (see Table 1). Three of 23 sites switched to digital teaching completely.

**Table 1:** Participating universities with offered teaching and exam formats. MC: Multiple choice, OSCE: Objective structured clinical examination

Department	Teaching format	Concept	Tool	Platform	Patient contact	Exam format	Concept	Platform
1	Lecture	Online	Recorded lecture	Online portal	No	MC	Presence	
	Seminar	Online	Video conference	Microsoft Teams	No			
	Bedside class	Hybrid	conference	Microsoft Teams	No			
	Voluntary class	Hybrid	conference	Microsoft Teams	No			
2	Bedside class	Presence	Patient examination		Yes	OSCE	Presence	
	Lecture	Online	Inverted Classroom Modell	Zoom	No	Oral exam	Hybrid	Zoom
	Seminar	Hybrid	Rounds		Yes	MC	Online	Moodle
	Voluntary class	Hybrid	Online work sheets	Zoom	No			
3	Lecture	Online	Recorded lecture	Online portal	No	Oral exam	Online	Online portal, Skype for business
	Bedside class	Presence		Online portal	No			
	Seminar	Online	Video conference	Skype for business	No			
	Voluntary class	Online	Video conference	Skype for business	No			
4	Lecture	Online	Recorded lecture	Online Portal	No	MC	Presence	
	Bedside class	Presence			Yes	OSCE	Online	Zoom
	Voluntary class	Presence			No			
5	Lecture	Online	Video conference	Zoom	No	MC-	Online	
	Bedside class	Hybrid	Video conference		Yes	OSCE	Presence	
	Seminar	Online	Live vs. Recorded		No	Oral exam	Hybrid	
6	Lecture	Online	Recorded lecture	Power Point	No	MC exam	Presence	
	Seminar	Online	Video conference	Webex	No			
	Bedside class	Online	Recorded lecture	Power Point	No			
	Case study	Online	Video conference	Webex	No			
7	Lecture	Online	Video conference	Microsoft Teams	No	Oral exam	Presence	
	Seminar	Online	Video conference	Microsoft Teams	No			
	Bedside class	Presence			Yes			
	Case study	Presence			No			
	Voluntary class	Presence						
8	Lecture	Online	Recorded lecture	Online portal	No	MC	Presence	
	Seminar	Online	Video conference	Online portal	No	Oral exam	Presence	
	Bedside class	Hybrid	Rounds	Online portal	Yes			
	Voluntary class	Hybrid	Video conference	Online portal	No			
9	Lecture	Online	Recorded lecture	Online portal	No	MC	Online	Moodle
	Seminar	Online	Video conference	Zoom	No	OSCE	Presence	
	Bedside class	Presence			Yes			
	Case study	Online	Video conference	Zoom	No			
	Voluntary class	Presence			No			
10	Lecture	Online	Podcast		No	MC	Online	Lime Survey
	Bedside class	Online	Podcast, demonstration videos		No			
	Case study	Hybrid	Video conference		No			
11	Lecture	Online	Recorded lecture	Moodle/Panopto	No	MC	Online	Moodle
	Seminar	Online	Video conference	Microsoft Teams	No			
	Bedside class	Presence			Yes			
	Case study	Online	Video conference	Microsoft Teams	No			
12	Lecture	Online	Recorded lecture		No	MC	Presence	
	Seminar	Online	Video conference	Bigbluebutton	No	OSCE	Presence	
	Bedside class	Hybrid			Yes			
	Case study	Hybrid			No			
13	Lecture	Online	Recorded lecture	Online portal	No	MC	Presence	
	Seminar	Online	Video conference	Online portal	No			
	Bedside class	Presence			Yes			
	Case study	Online	Video conference	Online portal	No			
	Voluntary class	Presence			No			
14	Lecture	Online	Live lecture	Zoom	No	MC	Online	
	Seminar	Online	Video conference	Zoom	No	Oral exam	Hybrid	Zoom
	Bedside class	Presence			Yes (Partially)			
15	Lecture	Hybrid	Live lecture	Online portal	No	MC	Presence	
	Bedside class	Presence	Examination practice	-	No			
	Bedside class	Presence	Rounds	-	Yes			
16	Lecture	Online	Live lecture	Zoom	No	MC	Presence	
	Seminar	Online	Video conference	Zoom	No	Oral exam	Presence	
	Bedside class	Presence			No	OSCE	Presence	
	Voluntary class	Online	Video conference	Zoom	No			
17	Lecture	Online	Recorded lecture	Zoom	No	MC	Presence	
	Seminar	Hybrid	Video conference	Zoom	No	Oral exam	Presence	
	Bedside class	Presence			No	OSCE	Presence	
	Case study	Presence			No			
	Voluntary class	Hybrid			No			
18	Lecture	Online	Recorded lecture	Online portal	No	MC	Presence	
	Bedside class	Online	Video conference	Online portal	No			
	Case study	Online	Video conference	Zoom	No			
19	Lecture	Online	Live lecture	Zoom	No	MC	Presence	
	Seminar	Presence			No	Oral exam	Presence	
	Case study	Online	Video conference	Zoom	No	OSCE	Online	Zoom
	Voluntary class	Presence			No			
20	Lecture	Online	Recorded lecture	Online portal	No	MC	Presence	
	Seminar	Presence	Video conference	Online portal	No	Oral exam	Presence	
	Bedside class	Presence			Yes			
	Voluntary class	Hybrid	Video conference	Online portal	No			
21	Lecture	Online	Recorded lecture	Moodle	No	MC	Hybrid	
	Seminar	Online	Video conference	Microsoft Teams	No			
	Bedside class	Hybrid	Video conference	Microsoft Teams	Yes (Partially)			
	Case study	Online	Video conference	Microsoft Teams	No			
22	Lecture	Online	Video conference	Webex	No	MC	Presence	
	Seminar	Online	Video conference	Webex	No	Oral exam	Presence	
	Bedside class	Hybrid		Webex	Yes			
	Voluntary class	Online	Video conference	Webex	No			
23	Lecture	Online	Recorded lecture	Zoom	No	MC	Presence	
	Seminar	Online	Video conference	Zoom	No			
	Case study	Online	Video conference	Zoom	No			
	Bedside class	Online	Video conference	Zoom	No			
	Voluntary class	Online	Video conference	Zoom	No			
24	Lecture	Online	Video conference	Online portal	No	MC	Presence	
	Seminar	Online	Video conference	Online portal	No	Oral exam	Presence	
	Case study	Hybrid	Video conference	Microsoft Teams	No	OSCE	Presence	
	Bedside class	Hybrid	Video conference	Microsoft Teams	No			
	Voluntary class	Hybrid	Video conference	Microsoft Teams	Yes			

### Digital teaching formats & tools

**Fig. 1:**
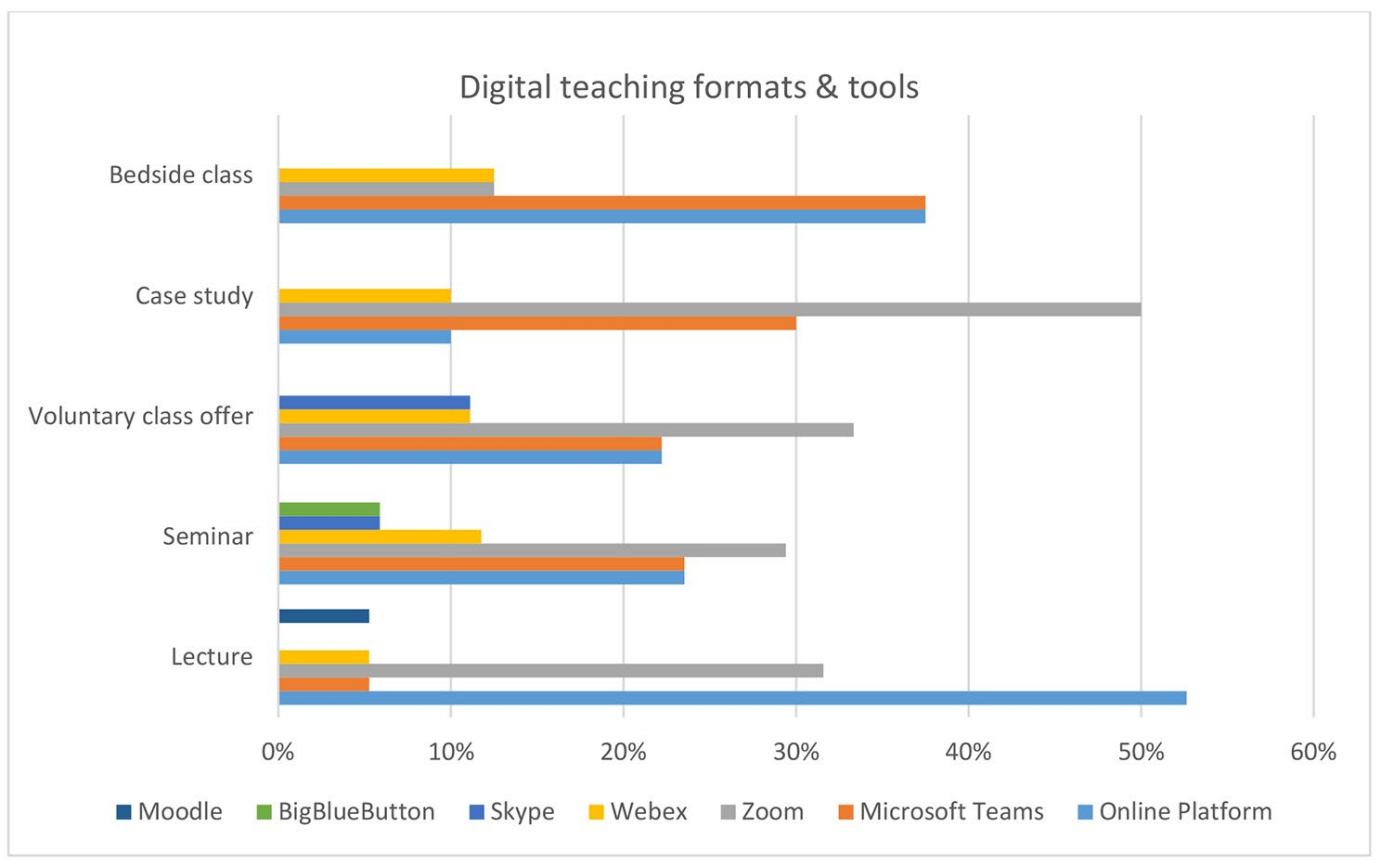
Digital teaching formats and used tools according to study participants (lectures n = 19; seminar n = 17; voluntary class offer n = 9; case study n = 12; bedside class n = 8)

While some teaching formats were almost completely switched to digital concepts, e.g. lectures and seminars, others like bed side classes were only transformed into hybrid concepts, allowing hands-on-training and patient contact of some extent. For the digital formats, differences in the used tools could be seen (see Fig. 1). The most commonly used platform for lectures were faculty specific platforms (53%), whereas seminars and voluntary classes also used Zoom and Microsoft teams.

### Performance review

Multiple choice exams were used by all universities, mostly in personal presence (17 of 22). This was enabled by hygiene concepts under state regulations: examinees were split into smaller groups, seated in larger lecture halls, exam questions were reduced to shorten the required time, all rooms were ventilated regularly, entrance and exit were separated, mouth-nose-coverage was mandatory, Covid-19 testing or full vaccination was required shortly before participating.

Oral exams were continued at 12 faculties, mostly in person (10 of 12). Objective Structured Clinical Examinations (OSCE) were carried out in person (6 of 8) as well as online (2 of 7). Only two departments decided to suspend this exam format.

### Local findings

In addition to the table above, this paragraph is supposed to focus on a couple of unique aspects found during our study.

This includes the offer for digital patient rounds and the live stream of emergency room management for medical students. Another university was able to report all their students to be fully vaccinated by the start of 2021, enabling more classroom teaching.

Other teaching concepts, such as additional voluntary classes were continued at 9 universities and were said to be an essential part in the education and commitment of students to the field of orthopaedics and trauma. One example would be the focused teaching in “Digital Health” at Charité university hospital in Berlin [14]. By implementing a digital health module for undergraduate medical students at Charité, “increased awareness for the importance and potential future impact of digital health on physicians’ work” could be shown [15].

### Potentials & pitfalls

**Fig. 2:**
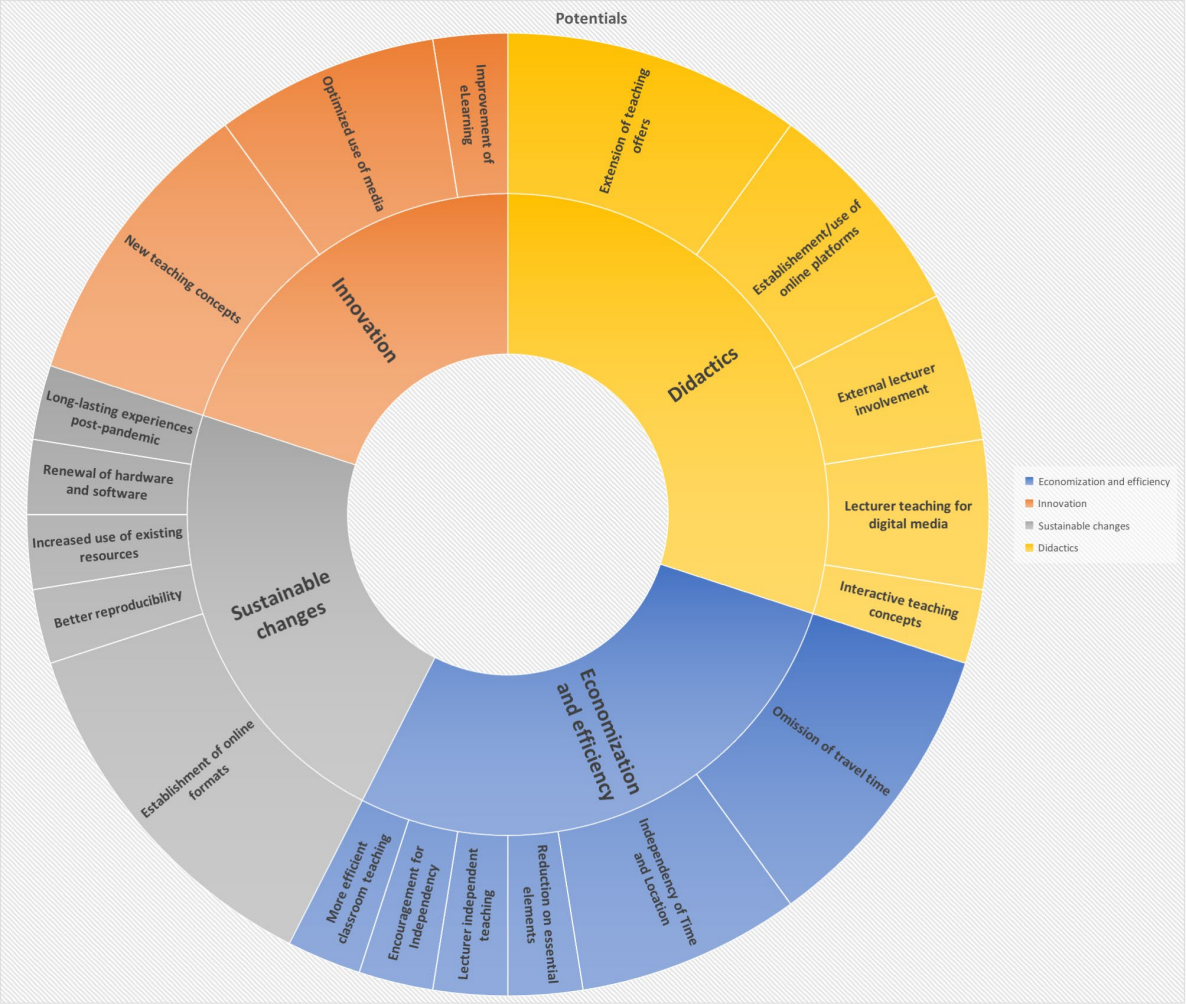
Potentials according to study participants from the department of Orthopaedics and/or Trauma (n = 19)

A frequent answer to the question of possible potentials arising from digital teaching was the increase in attendance explained by the omission of travel time as well as the new flexibility in time and location due to asynchronous teaching (see Fig. 2). Furthermore, several departments reported an increased consistency for quality and reproducibility in medical teaching. The involvement of external lecturers was suggested to improve the exchange between universities.

The necessity and support for more independent learning by students was mostly mentioned as a positive trait. Hybrid concepts were able to provide theoretical knowledge through digital teaching and increase the time for interactive practice during classroom teaching sessions.

**Fig. 3:**
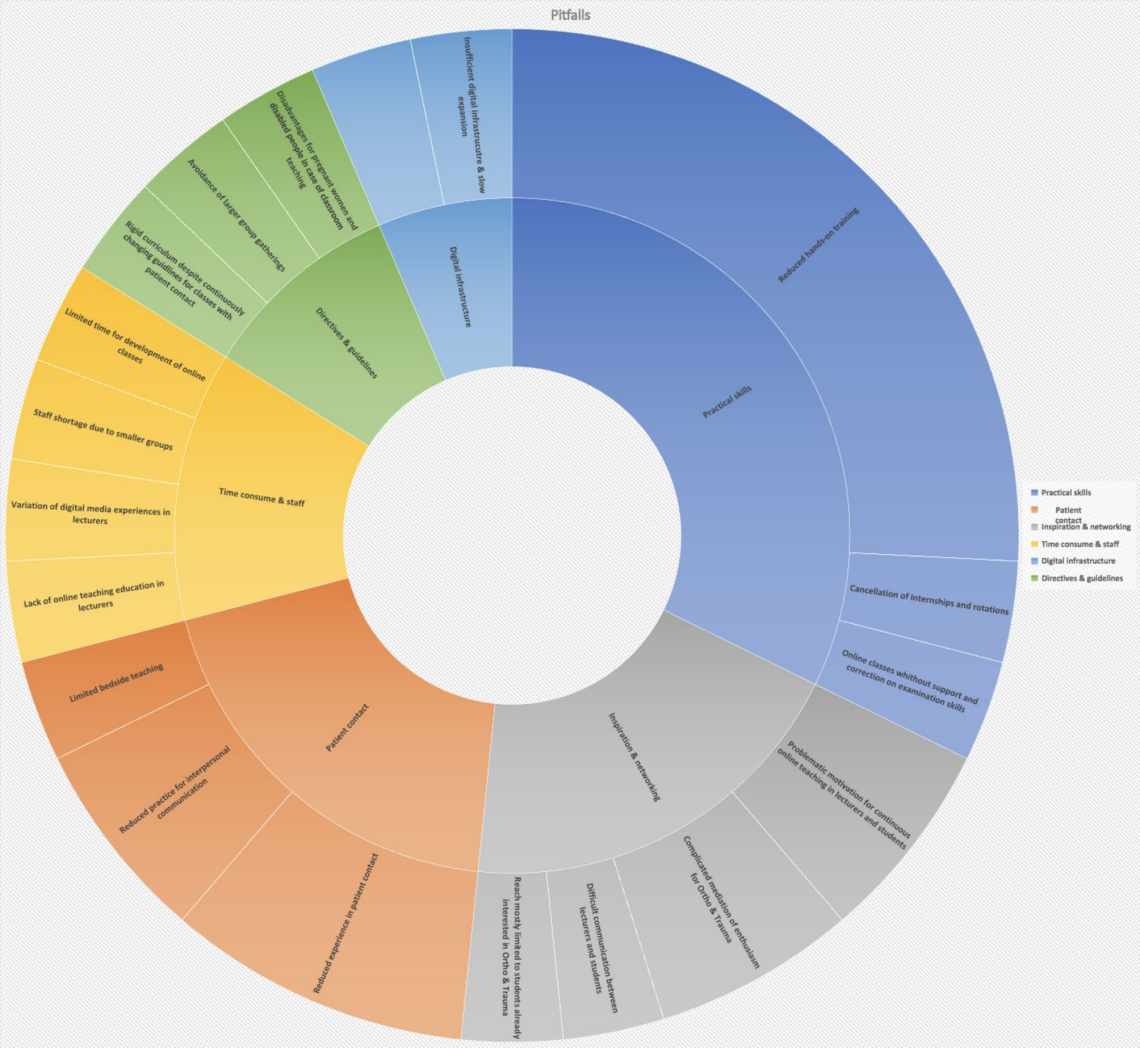
Pitfalls according to study participants from the department of Orthopaedics and/or Trauma (n = 19)

Nearly all participants of this study named the omission of hands-on practice to be the biggest challenge and a huge possible pitfall (see Fig. 3). This was mostly explained by the essential need for hands-on practice in Orthopaedics and Trauma.

Equally, basic conditions were criticised, such as lack of time to develop digital concepts and to transfer existing teaching concepts into digital classes, lack of infrastructure and its expansion for proper digital teaching, shortage of staff for classroom teaching due to the splitting of groups into several smaller groups and the omission of internships and rotations for medical students. Disadvantages concerning pregnant and disabled students in case of classroom teaching under special hygiene regulations by the state were also emphasized.

### Optimization proposals

**Fig. 4:**
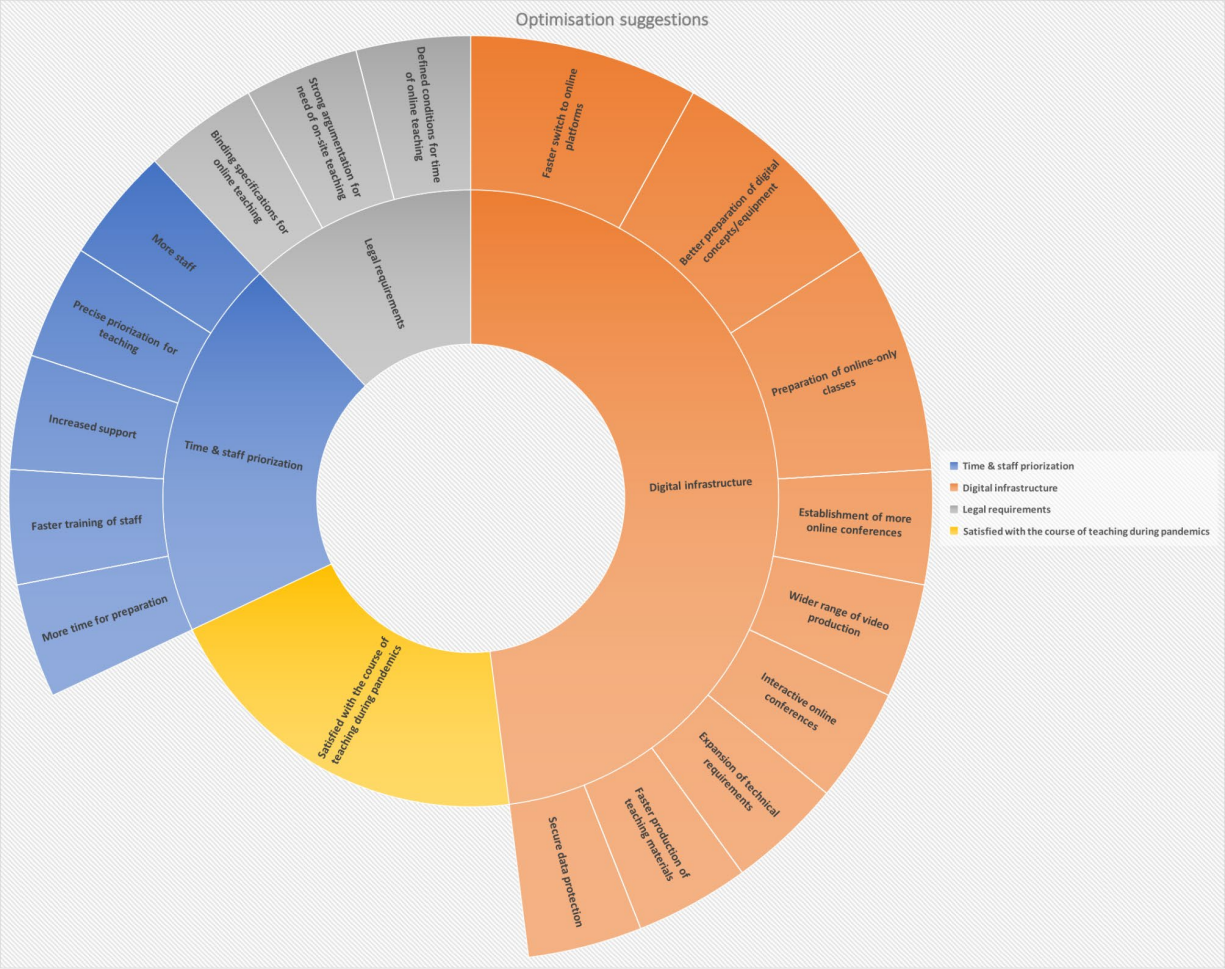
Optimisations suggestions according to study participants from the department of Orthopaedics and/or Trauma (n = 14)

In light of the mentioned challenges and pitfalls, 14 of 24 sites offered their suggestions on how to optimize the current medical teaching situation (see Fig. 4). Of the 14 replies to the question regarding optimization suggestions, 36% stated to be satisfied with the course of teaching during the pandemic without further elaboration or recommendations. More time and (educated) personnel to develop the teaching concepts and prepare their realization were the most basic demand to enable the production of qualitatively consistent teaching material provided for the students. Furthermore, several respondents demanded an update of the digital infrastructure provided by their department. At the same time the described the need of privacy policies regarding digital teaching to be defined more precisely, especially if it comes to the involvement of patients for digital classes.

## Discussion

Our survey showed that all universities that participated were confronted with transformation of teaching and study material in a remarkably short period of time. Since this process was subject to each university and/or department individually, a wide variety of digital teaching concepts for lectures, seminars and practical training, as well as the used platforms were found. The remaining classroom teaching was also subject to each department. While a few universities restrained from doing any classroom teaching at all, others tried to prioritize bedside teaching for higher semesters to enable practical experiences.

A specific challenge arises for students of the model degree program of medicine. This program was designed to emphasize patient contact starting on the first day of medical studies and to improve practical skills through experience [16]. Due to the lack of classroom and especially bedside teaching, earlier semesters are deprived of practical experiences. The resulting deficit in basic skills could be difficult to compensate later on, since the program is arranged modularly and supposed to work as a learning spiral. But not only students of this program could be affected, the time lost for practical experiences could probably affect all medical students.

Considering existing national and international literature, our results generally confirm the findings of other subjects and lecturers around the world [17–19].

Even though no other work explicitly focused on the subject of Orthopaedics and Trauma for this kind of topic regarding digital teaching, similar sources described pragmatic suggestions for solutions. This includes the usage of digital media to preserve a culture of interactive discussion between lecturers and students [20], the usage of pre-existing communication tools to train professional conversations with patients and acquire their case history [21], the continuation of knowledge transfer by digital recordings [22] or digitalization of medical exams [23]. General challenges, such as the ability to give individual feedback to students in online classes is discussed [24].

An advantage of online teaching could be the consistency in quality of knowledge transfer, e.g. in case of pre-recorded lectures available to all students. This includes the ability for students to repeat already watched as well as catch up with missed classes without depending on time and location. Also, lecturers could profit from this independence, easing the coordination of clinical work and teaching. The additional involvement of external lecturers could improve the professional exchange between universities and departments.

To completely overcome these didactic and organizational challenges, more time and experience is needed. Still, this paragraph will aim to provide an overview of promising concepts and potentials in Orthopaedics and Trauma teaching.

Students could be involved into telemedical consultations similar to the well-known bedside teaching concepts [18]. As soon as real patients are present, difficulties regarding data protection will arise and be of high importance to regulate.

Another interesting potential could be the usage of virtual reality (VR) and augmented reality (AR) to experience study material in new ways, e.g. anatomy [25].

In Orthopaedics VR has already been used for quite a while, enhancing practical skills and knowledge of students [26].

To enable good digital teaching, lecturers will be in need for adequate training on didactics in digital teaching. This includes knowledge on possibilities and limitations for each used online platform, basic conditions for privacy policy, and how to design digital exams.

Using previously named teaching materials, each class can be designed to be more attractive and exciting but also to appropriately convey knowledge to the students. A promising approach may be the hybridization of teaching formats, allowing students to prepare theoretical knowledge in larger groups beforehand and therefore more effectively using hands-on-time in smaller groups. Thus, also further reducing the required staff and time for smaller group classes.

Meeting the student’s needs for a deeper understanding of the field and therefore reinforcing their interest in the field of Orthopaedics and Trauma surgery by improved teaching formats may eventually lead to a closer attachment of students to Orthopaedics and Trauma and hopefully even in their application for residency in this field.

To train students on being digitally competent, it will also be essential to the field of Orthopaedics and Trauma to partake in the organization and teaching of classes. Considering the growing implementation of digital media and tools in our daily and work lives, it keeps gaining importance. Experiences and knowledge besides the “standard medical knowledge” will be helpful to future doctors [14] since they might use artificial intelligence and digitally-assisted systems to help with medical decision making [27].

### Limitations

Replies from the department of Orthopaedics and Trauma at 23 universities do not allow a generalization of our findings on teaching at all universities and departments in Germany. We decided to refuse a distinction between Orthopaedics and Trauma in order to create a general overview, even though they are still often organised and taught separately at German universities. Even before the start of the pandemic there were differences in the teaching of Orthopaedics and Trauma, meaning not all differences can be solely related to the pandemic.

The questionnaires were usually filled out by one person in charge of organising the teaching of their department at each medical university so some opinions could be subjective and not a proper reflection on the objective teaching situation.

This study only allows insight into the teacher’s perspective without regarding the opinions and immediate effects on the students themselves. Therefore, further studies may consider involving students for post-pandemic evaluation of digital teaching and training in Orthopaedics and Trauma surgery, as well as their personal experiences and academic performance during the pandemic.

Differences in the medical studies program, e.g. model degree vs. regular program, are designed differently and cannot necessarily be compared directly. Furthermore, the used definition of digital teaching is one of many and has to be kept in mind if comparisons to be made.

### Outlook

With increasing relevance of digitalization and the expected importance of digital competencies for doctors, teaching of medical students has to be adjusted as well. Modern and digital classes, individually adjusted to the students’ needs and interests will be essential. This provides attractive teaching of high quality and will additionally improve the engagement of students in the field of Orthopaedics and Tauma [28].

The exchange between universities and departments on experiences with old and new digital teaching concepts should be encouraged to provide overall improvement of teaching in this field. Larger concepts could be designed by several lecturers from different universities to achieve a high-quality result that can be distributed equally for digital training of traumatologists and orthopaedists. The peer-reviewed videos from the German working group on teaching (AG Lehre) serve as an example here. By showing general examination skills using patient models as well as 3D animations, the basic anatomy of different joints is explained [29].

To facilitate the exchange between universities it can be taken into consideration to use pre-existing platforms like the German Society for Orthopaedics and Trauma (DGOU) or the Working Group for Osteosynthesis (AO).

## Conclusion

One year into the pandemic, we can observe significant differences concerning the proportions of classroom and digital teaching for Orthopaedics and Trauma. Simultaneously there are major differences in the concepts used to create and eventually perform digital teaching. Especially Orthopaedics and Trauma were affected by the new regulations limiting classroom and bedside teaching with hands-on practice and patient contact. Since a complete suspense of classroom teaching was never mandatory, several universities developed hygiene concepts to enable some extent of practical experience for medical students. Still, patient contact was often limited to the higher semester and the organisation of classroom teaching was associated with the need for more lecturers and proper room capacities. Classes that could not be held in presence were transformed into a digital format over an amazingly short period of time even though all universities and departments had to organise this individually.

Despite all differences in digital teaching mentioned above, some similarities could be observed: the lack of time and personnel to generate adequate teaching material was reported as the leading challenge by all participants of this study. At the same time most universities saw potential in the use of digital formats, since it could offer consistency in teaching quality and independence in time and location for students as well as lecturers.

Therefore, the use of digital teaching formats is, without doubt, essential to the future of medical studies and has gained importance not just due to the pandemic, but due to the general digitalization of our world. By implementing and improving digital teaching and possible concepts today and post-pandemic, important digital competencies can be encouraged in students early on. Realizing the potentials of digital teaching, it can and should be used complementary to the practical training of medical students. However, it can probably never completely replace classroom and bedside teaching. As one of the main pillars in the field of medical studies is the direct communication with patient, it contributes to the personal and professional growth of all students aspiring to become doctors.

## Electronic supplementary material

Below is the link to the electronic supplementary material.

Supplementary Material 1

## Data Availability

The datasets used and/or analyzed during the current study are available from the corresponding author on reasonable request.

## Abbreviations

AO: Working group for osteosynthesis
AR: Augmented reality
DGOU: German association for Orthopaedics & Trauma
MC: Multiple choice
OSCE: Objective structured clinical examination
VR: Virtual reality
