# Supplementary material for: Medical studies in times of a pandemic – concepts of digital teaching for Orthopaedics and Trauma at german universities
Source: BMC Med Educ. 2023 Apr 18;23:257. doi: 10.1186/s12909-023-04213-4 (PMC10112299; doi:10.1186/s12909-023-04213-4)
Supplement: Supplementary file 1 — Supplementary Material 1 [file 12909_2023_4213_MOESM1_ESM.docx]

1. Which medical university in Germany are you replying for?
2. What kind of medical program does your university offer? (multiple choices possible)
   1. Standard course („Regelstudiengang“)
   2. Reform course („Reformstudiengang“)
   3. Model degree program („Modellstudiengang“)
3. Teaching in Orthopaedics and Trauma is offered in which semesters?

For the following questions 4-6, please fill out the table below.

.

1. Which teaching formats were used? Please choose all that are applicable.
2. Please indicate the corresponding concepts used for each teaching format (Online, presence, hybrid).
3. Which platforms were used for online teaching (MS Teams, Zoom, Skype, Online portal etc.)?

| **Teaching format** | **Conducted during the pandemic?**  **(yes/no)** | **Concept (Online/presence/hybrid)** | **Platform**  **(MS Teams, Zoom, Skype, Online portal etc.)** |
| --- | --- | --- | --- |
| Lecture |  |  |  |
| Seminar |  |  |  |
| Bedside class |  |  |  |
| Case study |  |  |  |
| Voluntary class |  |  |  |
| Others: |  |  |  |

1. Was patient contact at all possible for students?
   1. If yes, for which formats?
   2. What kind of regulations applied for teaching formats with patient contact (specific hygiene concepts etc.)?

For the following questions 8 -10, please fill out the table below.

1. Which teaching formats were used? Please choose all that are applicable.
2. Please indicate the corresponding concepts used for each teaching format (Online, presence, hybrid).
3. Which platforms were used for online teaching (MS Teams, Zoom, Skype, Online portal etc.)?

| **Exam format** | **Concept (Online/presence/hybrid)** | **Platform**  **(Online portal, LimeSurvey etc.)** |
| --- | --- | --- |
| Multiple choice |  |  |
| Oral exam |  |  |
| Objective structured clinical examination |  |  |
| Others: |  |  |

1. What kind of regulations applied for exam formats in presence (specific hygiene concepts etc.)?
2. Were additional voluntary classes for Orthopaedics and Trauma offered during the pandemic?
   1. Yes/no
   2. If yes, what kind of voluntary classes?
3. Which local „particularities“ would describe the teaching concepts at your university during the pandemic?
4. Please describe possible potentials arising from the inevitable transformation/change of teaching formats in Orthopaedics and Trauma during the pandemic.
5. What kind of possible pitfalls do you identify for teaching in Orthopaedics and Trauma during the pandemic?
6. Which teaching formats were especially challenging or not possible for Orthopaedics and Trauma during the pandemic?
7. In retrospect, would you change anything about the organisation or conduction of your teaching during the pandemic? If so, how?
